# Supplementary material for: Can a history of crop rotations improve the prediction of soil organic carbon in the Andes? integrating machine learning multi-annual crop classification as a proxy of soil management
Source: PLoS One. 2026 Jul 16;21(7):e0353966. doi: 10.1371/journal.pone.0353966 (PMC13375141; doi:10.1371/journal.pone.0353966)
Supplement: S1 Table — Classification features for multi-year cropland classification using Sentinel-2 imagery A and B time series (S2A, S2B) from the Harmonized Sentinel-2 Multispectral Instrument (MSI). Blue, green, red, near-infrared (NIR), shortwave infrared 1 (SWIR1), and shortwave infrared 2 (SWIR2) are surface reflectance values from Band 2 (blue, 496.6 nm (S2A)/492.1 nm (S2B)), Band 3 (green, 560 nm (S2A)/559 nm (S2B)), Band 4 (red, 664.5 nm (S2A)/665 nm (S2B)), Band 8 (NIR, 835.1 nm (S2A)/833 nm (S2B)), Band 11 (SWIR1, 1613.7 nm (S2A)/1610.4 nm (S2B)), and Band 12 (SWIR2, 2202.4 nm (S2A)/2185.7 nm (S2B)). f = 0.05. (DOCX) [file pone.0353966.s001.docx]

|  | Index/Parameter Definition | Acronym/Formula |
| --- | --- | --- |
| Phenology Gaussian Features | Midpoint of the crop growth curve | µ |
|  | Vegetative period | σ |
| Vegetation Indices | Bare Soil Index (BSI) evaluated at µ | $BSI =\frac{\left( SWIR2+R \right)- (NIR+B)}{\left( SWIR2+R \right) + (NIR+B)}$ |
|  | Normalized Difference Vegetation Index (NDVI) evaluated at µ ($\mathrm{NDVI}_{MAX}$) | $NDVI =\frac{NIR-R}{NIR + R}$ |
|  | Specific Leaf Area Vegetation Index (SLAVI) evaluated at µ | $SLAVI=\frac{NIR}{RED+ SWIR1}+f$ |
|  | Normalized Burn Ratio 2 (NBR2) evaluated at µ | $NBR2= \frac{SWIR1-SWIR2}{SWIR1 + SWIR2}$ |
|  | Normalized Difference Moisture Index (NDMI) evaluated at µ | $\mathrm{NDMI}= \frac{NIR-SWIR2}{NIR+ SWIR2}$ |
| Dynamic Time Warping (DTW) Features | Cropland cluster groups from DTW hard clustering | DTW_POTATO_, DTW_LUPIN_, DTW_FALLOW_, DTW_BEANS_, DTW_BARLEY_, DTW_PASTURE_, DTW_OATS_ |
|  | Cropland cluster groups from DTW fuzzy clustering | DTW_FUZZ_ |
