# Supplementary material for: Can a history of crop rotations improve the prediction of soil organic carbon in the Andes? integrating machine learning multi-annual crop classification as a proxy of soil management
Source: PLoS One. 2026 Jul 16;21(7):e0353966. doi: 10.1371/journal.pone.0353966 (PMC13375141; doi:10.1371/journal.pone.0353966)
Supplement: Attachment — Submitted filename: Response to reviewers_PONE-D-25-56000.docx [file pone.0353966.s007.docx]

**Response to comments made by reviewers to the manuscript PONE-D-25-56000 entitled: Can a history of crop rotations improve the prediction of soil organic carbon in the Andes? Integrating machine learning multi-annual crop classification as a proxy of soil management**

We would like to thank you for the thorough and very constructive review, which we found agree with the recommendations and have changed the corresponding parts of the manuscript accordingly. All the editor and reviewer’s comments were addressed and responded in the same order, as follows:

**REPLIES TO JOURNAL REQUIREMENTS:**

1.Please ensure that your manuscript meets PLOS ONE's style requirements, including those for file naming. The PLOS ONE style templates can be found at

<https://journals.plos.org/plosone/s/file?id=wjVg/PLOSOne_formatting_sample_main_body.pdf> and

<https://journals.plos.org/plosone/s/file?id=ba62/PLOSOne_formatting_sample_title_authors_affiliations.pdf>

Our response:

Thank you for your comment. We have used the PLOS ONE LaTeX template and confirm that we have followed the style requirements, including those for the captions of figures in the manuscript and the supplementary figures.

**2.** In your Methods section, please provide additional information regarding the permits you obtained for the work. Please ensure you have included the full name of the authority that approved the field site access and, if no permits were required, a brief statement explaining why.

Our response:

This study was conducted in the district of Chugay, located in the Andean region of the La Libertad department in Peru, with support from Asociación Pataz (https://asociacionpataz.org.pe/), an organization whose mission is to promote sustainable development opportunities for vulnerable communities. One of the co-authors of this paper is a staff member of Asociación Pataz, which facilitated coordination with local stakeholders. Through this organization, we obtained the necessary permissions from the local authorities of Chugay to access farmers’ fields.

In Annex 1, at the end of the document, we present the permits granted by the Mayor of the District of Chugay for this study.

The following changes have been introduced to the manuscript:

**Page 3, Material and Methods section, line 114 to 118:**

Cropland cover information of 189 fields was collected in 2022 and 2024 through field visits (Rinza, 2022) and farmer surveys with prior authorization from the District Municipality of Chugay, facilitated by Asociación Pataz (https://asociacionpataz.org.pe/), an organization whose mission is to promote sustainable development opportunities in vulnerable communities.

**3.** Thank you for stating the following financial disclosure:

“This work was supported, in whole or in part, by the Bill and Melinda Gates Foundation (BMGF; grant number OPP1070785), the project Sustainable Farming CGIAR Science Program, and the Deutsche Gesellschaft für Internationale Zusammenarbeit (GIZ) through the fund for the promotion of innovation in agriculture (i4Ag; Agreement 543 N◦: 81275993). It was also conducted as part of the CGIAR Initiative on Excellence in Agronomy, now integrated into the CGIAR Sustainable Farming Science Program. CGIAR research is supported by contributions to the CGIAR Trust Fund. CGIAR is a global research partnership for a food-secure future dedicated to transforming food, land, and water systems in a climate crisis.”

Please state what role the funders took in the study. If the funders had no role, please state: "The funders had no role in study design, data collection and analysis, decision to publish, or preparation of the manuscript."

If this statement is not correct you must amend it as needed.

Please include this amended Role of Funder statement in your cover letter; we will change the online submission form on your behalf.

Our response:

We have removed the support for Excellence in Agronomy from the Acknowledgments section. Accordingly, the revised Acknowledgments section reads as follows:

“This work was supported, in whole or in part, by the Bill and Melinda Gates Foundation (BMGF; grant number OPP1070785), the CGIAR Sustainable Farming Science Program, and the Deutsche Gesellschaft für Internationale Zusammenarbeit (GIZ) through the fund for the promotion of innovation in Agriculture (i4Ag; Agreement 543 No. 81275993). CGIAR is a global research partnership for a food-secure future dedicated to transforming food, land, and water systems in a climate crisis.”

Regarding the role of the funders:

- The CGIAR Sustainable Farming Science Program provided support for laboratory analyses, as well as personnel salaries and time.
- The Deutsche Gesellschaft für Internationale Zusammenarbeit (GIZ), through the i4Ag fund (Agreement No. 81275993), supported field travel and contributed to personnel time.
- The Bill and Melinda Gates Foundation (BMGF; grant number OPP1070785) provided support through equipment acquired during the implementation of the project, which was subsequently used in this study, including GPS devices, RGB cameras, and field sampling tools.

**4.** Thank you for stating the following in your Competing Interests section: “NO”

Please complete your Competing Interests on the online submission form to state any **Competing Interests**. If you have no competing interests, please state "The authors have declared that no competing interests exist.", as detailed online in our guide for authors at

<http://journals.plos.org/plosone/s/submit-now>

This information should be included in your cover letter; we will change the online submission form on your behalf.

Our response:

Thank you for your comment. Please note that: “The authors have declared that no competing interests exist”

**5.** Please note that your Data Availability Statement is currently missing the repository name and/or the DOI/accession number of each dataset OR a direct link to access each database. If your manuscript is accepted for publication, you will be asked to provide these details on a very short timeline. We therefore suggest that you provide this information now, though we will not hold up the peer review process if you are unable.

Our response:

Thank you for your comment. We have included the following dataset in the paper:

**Page 5, Material and Methods section, lines 176 to 178:**

The resulting processed data, which provides the time-series data cube of Sentinel-2 from 2019 to 2024 imagery and crop labels, is openly accessible at <https://zenodo.org/records/19949051>.

**Page 8, Material and Methods section, lines 308 to 312:**

The soil dataset supporting the findings of this study, which includes the physical-chemical properties and soil organic carbon (SOC) measurements for the 189 sampled fields in the Northern Highlands of Peru, is publicly available through the International Potato Center (CIP) Dataverse at: https://doi.org/10.21223/P3/RHE9QI.

**6.** We note that Figure 1 and 5 in your submission contain map/satellite images which may be copyrighted. All PLOS content is published under the Creative Commons Attribution License (CC BY 4.0), which means that the manuscript, images, and Supporting Information files will be freely available online, and any third party is permitted to access, download, copy, distribute, and use these materials in any way, even commercially, with proper attribution. For these reasons, we cannot publish previously copyrighted maps or satellite images created using proprietary data, such as Google software (Google Maps, Street View, and Earth). For more information, see our copyright guidelines: <http://journals.plos.org/plosone/s/licenses-and-copyright>.

We require you to either (1) present written permission from the copyright holder to publish these figures specifically under the CC BY 4.0 license, or (2) remove the figures from your submission:

1. You may seek permission from the original copyright holder of Figure 1 and 5 to publish the content specifically under the CC BY 4.0 license.

 We recommend that you contact the original copyright holder with the Content Permission Form (<http://journals.plos.org/plosone/s/file?id=7c09/content-permission-form.pdf>) and the following text:

“I request permission for the open-access journal PLOS ONE to publish XXX under the Creative Commons Attribution License (CCAL) CC BY 4.0 (<http://creativecommons.org/licenses/by/4.0/> ). Please be aware that this license allows unrestricted use and distribution, even commercially, by third parties. Please reply and provide explicit written permission to publish XXX under a CC BY license and complete the attached form.”

Please upload the completed Content Permission Form or other proof of granted permissions as an "Other" file with your submission.

 In the figure caption of the copyrighted figure, please include the following text: **“Reprinted from [ref] under a CC BY license, with permission from [name of publisher], original copyright [original copyright year].”**

Our response:

Thank you for your comment. Figures 1 and 5 include maps derived from the Sentinel-2 and ALOS PALSAR platforms. The corresponding figure captions have been revised accordingly:

**Page 3, Material and Methods section, Line 89:**

Fig 1: The study area. The study area (indicated by a rectangle over a Sentinel-2 scene from 17/08/2019) is situated in the Chugay District, La Libertad Department, Peru (A). Red triangles denote soil organic carbon sampling points, while green patches depict cropland fields in the study area, shown over a Digital Elevation Model from ALOS PALSAR data distributed by Alaska Satellite Facility (B). Monthly mean minimum (blue) and maximum (red) temperatures, and monthly accumulated precipitation (sky-blue bars) for the 1981--2016 period, derived from PISCO datasets (C).

**Page 11, Results section, line 405:**

Fig 5. Crop rotation features. Spatial distribution of the features generated by the multi-year cropland classification model from 2019 to 2022, including both surveyed and digitized fields for the study area croplands, overlaid on Sentinel-2 imagery from the European Union Copernicus Programme.

7. We note that Figure S1 in your submission contain copyrighted images. All PLOS content is published under the Creative Commons Attribution License (CC BY 4.0), which means that the manuscript, images, and Supporting Information files will be freely available online, and any third party is permitted to access, download, copy, distribute, and use these materials in any way, even commercially, with proper attribution. For more information, see our copyright guidelines: <http://journals.plos.org/plosone/s/licenses-and-copyright> .

We require you to either (1) present written permission from the copyright holder to publish these figures specifically under the CC BY 4.0 license, or (2) remove the figures from your submission:

1. You may seek permission from the original copyright holder of Figure S1 to publish the content specifically under the CC BY 4.0 license.

We recommend that you contact the original copyright holder with the Content Permission Form (<http://journals.plos.org/plosone/s/file?id=7c09/content-permission-form.pdf>) and the following text:

“I request permission for the open-access journal PLOS ONE to publish XXX under the Creative Commons Attribution License (CCAL) CC BY 4.0

(<http://creativecommons.org/licenses/by/4.0/>). Please be aware that this license allows unrestricted use and distribution, even commercially, by third parties. Please reply and provide explicit written permission to publish XXX under a CC BY license and complete the attached form.”

Please upload the completed Content Permission Form or other proof of granted permissions as an "Other" file with your submission.

In the figure caption of the copyrighted figure, please include the following text: “Reprinted from [ref] under a CC BY license, with permission from [name of publisher], original copyright [original copyright year].”

2. If you are unable to obtain permission from the original copyright holder to publish these figures under the CC BY 4.0 license or if the copyright holder’s requirements are incompatible with the CC BY 4.0 license, please either i) remove the figure or ii) supply a replacement figure that complies with the CC BY 4.0 license. Please check copyright information on all replacement figures and update the figure caption with source information. If applicable, please specify in the figure caption text when a figure is similar but not identical to the original image and is therefore for illustrative purposes only.

Our response:

Thank you for your comment. Figure S1 consisted of photographs taken by one of the co-authors; therefore, no permission issues arose. However, in response to the reviewer’s suggestion to remove less relevant figures, we will remove Supplementary Figure S1.

**8**. If the reviewer comments include a recommendation to cite specific previously published works, please review and evaluate these publications to determine whether they are relevant and should be cited. There is no requirement to cite these works unless the editor has indicated otherwise.

Our response:

Thank you for your comment. The reviewer did not make this recommendation.

**9**.Please review your reference list to ensure that it is complete and correct. If you have cited papers that have been retracted, please include the rationale for doing so in the manuscript text, or remove these references and replace them with relevant current references. Any changes to the reference list should be mentioned in the rebuttal letter that accompanies your revised manuscript. If you need to cite a retracted article, indicate the article’s retracted status in the References list and also include a citation and full reference for the retraction notice.

Thank you for this comment. We have carefully reviewed the reference list for completeness and consistency with the manuscript content. Two references originally cited as a preprint and a dataset have been replaced with their corresponding peer-reviewed journal articles:

Originally cited as preprint:

Briceño P, Ninanya J, Seminario JF, Otiniano R, Rinza J, Mestanza C, et al. Can regenerative agriculture in Andean potato farming enhance productivity, profitability, and carbon sequestration while reducing carbon footprint? A case study from Northern Peru. SSRN Scholarly Paper No. 5347153. Social Science Research Network; 2025.

https://doi.org/10.2139/ssrn.5347153

Corrected version:

Briceño P, Ninanya J, Seminario JF, Otiniano R, Rinza J, Mestanza C, et al. Can regenerative agriculture enhance productivity, profitability, and reduce carbon emissions? A case study in Andean potato farming. European Journal of Agronomy. 2026;175:128020.

https://doi.org/10.1016/j.eja.2026.128020

Originally cited as dataset:

Carbajal M, Ramírez D, Turin C, Schaeffer S, Konkel J, Ninanya J, et al. From rangelands to cropland, land-use change and its impact on soil organic carbon variables in Peruvian Andean highlands: a machine learning modeling approach. International Potato Center; 2024.

https://doi.org/10.21223/VVIFL9

Corrected version:

Carbajal M, Ramírez DA, Turin C, Schaeffer SM, Konkel J, Ninanya J, et al. From rangelands to cropland, land-use change and its impact on soil organic carbon variables in Peruvian Andean highlands: a machine learning modeling approach. Ecosystems. 2024;27(7):899–917.

https://doi.org/10.1007/s10021-024-00928-7

In addition, one citation included in the original manuscript has been removed due to duplication, and another has been removed to address the reviewer’s comments and ensure consistency with the revised manuscript.

Removed due to duplication:

De Vroey M, Radoux J, Defourny P. Classifying sub-parcel grassland management practices by optical and microwave remote sensing. Remote Sensing. 2022;15(1):181.

https://doi.org/10.3390/rs15010181

Retracted citation:

Wu Y, Davis EC, Sohngen BL. Crop rotation and the impact on soil carbon in the U.S. Corn Belt. Carbon Balance and Management. 2025;20(1):6. https://doi.org/10.1186/s13021-025-00293-5

Finally, we included a relevant peer-reviewed reference to further support the role of agricultural management practices in soil organic carbon dynamics.

López I, Losada R, Hedlund K, Haddaway NR, Sahlin U, Jackson LE, Kätterer T, et al. Synergistic effects of multiple “good agricultural practices” for promoting organic carbon in soils: a systematic review of long-term experiments. Ambio. 2025;54(11):1715–1728.

https://doi.org/10.1007/s13280-025-02188-8

**REPLIES TO REVIEWER’S COMMENTS**

**Reviewer Comment 1:** The survey data covers the period from 2019 to 2022, while the remote sensing data spans from 2022 to 2024. How do you plan to integrate these datasets without introducing errors?

Our response:

The authors thank the reviewer for the comment. Soil sampling was conducted in 2022 across 189 cropland fields. In 2024, we revisited these fields and conducted a farmer survey to identify the crops grown, including fallow periods between 2022 and 2024. Using this information, we trained a crop classification model based on Sentinel-2 time series, structured according to two cropping seasons per year. Once trained, the model was used to estimate the crops grown in the 189 sampled fields for the 2019–2021 period, thereby reconstructing crop histories from 2019 to 2022.

This reconstruction introduces some uncertainty, as the prediction of crops for 2019–2021 required defining the start and end of the main and secondary growing seasons to extract the corresponding image time series. The onset of the main season was estimated using average precipitation data from the WorldClim repository, while the end of the main season and the start and end of the secondary seasons were determined based on average seasonal patterns derived from data spanning 2021 to 2024.

The following correction has been made to the manuscript:

**Page 7, Material and methods section, line 234 to 240:**

To predict the 2019–2022 CR using a model trained on the 2022–2024 field monitoring period without introducing temporal bias, we incorporated seasonal (MS, SS) indicator features into the model, as described in the labeling processing section. These features used windows defined by WorldClim precipitation averages and observed seasonal sowing and harvesting patterns. By establishing these consistent seasonal boundaries, the model could reliably project seasonal crop classifications back to the 2019–2022 period.

**Page 7, Material and methods section, lines 271 to 280:**

To integrate the spatially explicit, seasonally predicted crop type into an extended CR (2019–2022) dataset that could be meaningfully linked to SOC, we derived a 'cropping frequency' feature for each crop field. Integrating crop sequence information facilitated the incorporation of CR history into our SOC modeling framework. Consequently, cropping frequency was derived from the extended CR to quantify the prevalence of each crop type throughout the 2019–2022 campaigns. This approach enabled the integration of multi-year remote-sensing classifications as a predictive feature for measured SOC during the 2022 sampling campaign.

**Reviewer Comment 2:** You only used data from 2019. Please ensure that the term “historical” is used appropriately.

Our response:

The authors thank the reviewer for the comment. The following correction has been made to the manuscript:

**Page 5, Material and Methods section, lines 158 to 159:**

These cropland labels generally span several seasons within the same field, thus providing a reliable multi-season temporal record.

**Page 16, Conclusion section, lines 619 to 620:**

To our knowledge, this study is the first to estimate multi-season, field-scale crop rotations and incorporate them as predictors in SOC modeling in the Andes.

**Reviewer Comment 3:** The experiments have distinct purposes: one focuses on remote sensing classification, while the other focuses on soil property estimation. Can you integrate these two aspects more smoothly?

Our response:

The authors thank the reviewer for the comment. The following correction has been made to the manuscript to achieve your suggestion:

**Page 1, Abstract section:**

Crop rotation (CR) history was linked to ground-truthed soil data via a multi-year crop classification model trained on data from 534 fields across 2022–2024.

**Page 2 and 3, Introduction section, lines 45 to 67:**

However, the degree and variability of SOC accumulation in response to CR, along with the underlying factors driving this response, remain uncertain (Liu, 2022; Zou, 2015; Zou, 2018). This uncertainty stems from the complex interactions among influential factors—such as climate, soil properties, and agronomic practices—that often make predicting SOC responses to individual variables difficult (Liu, 2022). In specific contexts, inherent environmental variables and specific soil covariates may exert a significantly stronger influence on SOC stock variations than crop management history (Kellner, 2025; Liu, 2022; McBratney, 2003). Nevertheless, the reported importance of soil properties varies across scales (Liu, 2022). While climate covariates often outweigh soil predictors in broad regional or global studies like SoilGrids (Heuvelink, 2021; Sothe, 2022; Hengl, 2017), local-scale studies highlight the dominant explanatory role of soil texture. Because carbon accumulation is ultimately modulated by the interplay between clay concentration, land use, and climate (Lamichhane, 2019; Wiesmeier, 2019), site-specific analyses are essential to determine whether management-driven signals can be identified above the dominant influence of inherent soil characteristics.

Information about CR can be gathered from multi-annual crop inventories, government maps, surveys, and, more recently, earth observation (EO) technologies. Using satellite image time series has emerged as a promising approach for crop type classification in remote sensing. It captures spectral-temporal profiles from multispectral images taken throughout the growing season, reflecting crop phenology and seasonal variation. This approach enhances classification accuracy by extracting unique spectral-temporal features for various crops (Garnot & Landrieu, 2020; Peña & Brenning, 2015; Rustowicz et al., 2019). However, a notable gap remains in knowledge regarding the integration of soil management into agricultural remote sensing frameworks.

**Page 3, Introduction section (study objectives), lines 71 to 81:**

The specific objectives of the study were to: i) develop a phenological-based multi-year (2019–2023) cropland classification model integrating Sentinel-2 time series; ii) evaluate the predictive power of remote sensing-derived CR features for SOC estimation against traditional climatology and soil properties and assess the relative importance of these feature groups. The working hypothesis was that spatially explicit CR features, captured through multi-temporal classification would significantly enhance SOC prediction in heterogeneous Andean landscapes—offering a viable modeling alternative even in the absence of ground-based soil features.

**Reviewer Comment 4:** The results indicate that excluding crop rotation history yielded the highest performance. This seems contradictory to the rationale stated in the introduction section. Is there any misinterpretation? Do you also consider fallow periods as part of rotation?

Our response:

The authors thank the reviewer for the comment. Yes, our study considers fallow periods as part of crop rotations. Crop rotations are recognized as beneficial management practices that help maintain soil health in the Andes. In particular, higher frequencies of fallow periods are generally associated with healthier soils and higher SOC contents. However, previous studies have shown that crop rotation cycles of 8–10 years are typically required to observe measurable effects on SOC.

In this study, we consider a 4-year crop rotation period, including two years derived from farmer surveys and two years estimated through remote sensing. This relatively short time span may limit the ability to derive robust features for explaining SOC variability. Nevertheless, fallow frequency emerged as a relevant feature in explaining SOC variability in our results.

The following correction has been made to the manuscript:

**Page 14, Discussion section, lines 518 to 526:**

However, the statistical dominance of soil properties over CR features in this model may be attributed to two factors. First, soil properties such as clay content are directly related to the physical stabilization of SOC (Zou, 2018), consistently ranking as primary predictors in high-resolution models. Second, while CR impacts SOC dynamics, measurable changes in carbon stocks typically require rotation cycles of 8–10 years to manifest (King, 2018; Liu, 2022), a longer horizon than the modeling period. Nevertheless, the positive impact of reduced soil disturbance through permanent cover rather than annual cropping (Chen, 2022) remains evident.

**Page 5, Material and Methods section, lines 155 to 157:**

Thus, fallow was included as a core component of the CR definition, as higher frequencies of covered fallow periods are generally associated with increased SOC contents (Lopez-ILosada, 2025; DeHaan, 2010).

**Reviewer Comment** 5: You need some map to present the results of crop classification, crop rotation and soil property. In this regard, you could remove some figures that are less relevant.

Our response:

The authors thank the reviewer for the comment. The following changes have been made to the manuscript:

A new comprehensive map illustrating crop classification and crop rotation dynamics has been incorporated into the Results section. To prioritize the presentation of the crop history maps, Figure 4 (Classification of NDVI Time Series Using Dynamic Time Warping (DTW)) has been moved to the Supporting Information and replaced with the new crop classification maps.

**Page 10, Results section, lines 382 to 384:**

The resulting classification outputs enabled the identification of spatial and temporal crop rotation patterns across MS and SS during the 2019–2023 study period (Fig. 4).

**Page 10, Results section, line 381:**

Fig 4. Crop rotation patterns (2019–2023)**.** Spatial distribution of crop classification outputs across main and secondary growing seasons in a subarea of the study region, overlaid on Sentinel-2 RGB imagery from the Copernicus Programme. Colors represent the classified crop types and illustrate field-level crop rotation dynamics between 2019 and 2023.

Additionally, the previous Supplementary Figure S1 (Modeled crop types in the Chugay study area) has been removed, and the supplementary figures have been reorganized as follows:

- S1 Fig. Boxplots of NDVI-derived phenological parameters. (A) Maximum Normalized Difference Vegetation Index (NDVI) value at peak canopy cover time (NDVIMAX), (B) time to reach peak growth (*μ*), and (C) full width at half maximum (*σ*), estimated from Gaussian functions fitted to Sentinel-2 NDVI time series for each cropland type.
- S2 Fig. Classification of NDVI time series using Dynamic Time Warping (DTW). The width of the violin diagrams represents the contribution of the corresponding cluster for the characterization of each crop.
- S3 Fig. Confusion matrix for the test set. Test set corresponds to 30% of the full dataset (n = 788 instances).

**ANNEX 1:** Authorization for field access, soil sampling, and farmer surveys.
